# Editorial: Toward a Science of Complex Experiences

**DOI:** 10.3389/fpsyg.2021.775149

**Published:** 2021-11-30

**Authors:** Alice Chirico, Andrea Gaggioli

**Affiliations:** ^1^Department of Psychology, Università Cattolica del Sacro Cuore, Milano, Italy; ^2^ATN-P Lab, Istituto Auxologico Italiano, Milan, Italy

**Keywords:** emotion, complex experience, complexity, experience, paradox, novel

Complexity has been always a part of an individual's life under different guises. However, it has always been hard to provide a clear definition of what complexity really is. For instance, in the field of science, complexity has been defined in terms of systems [vol. 284, Issue 5411, pp. 1-212]. A system can be deemed as complex when multiple interactions occur among different components or when the system evolves over time, where a recognizable structure is still present but varies in different ways in response to small perturbations or by being sensitive to initial conditions. This definition, as well as the concept of a system, can also be applied to human experience (e.g., Cipresso, 2010), whose unfolding takes place through the interactions of specific cognitive, perceptual, and emotional components bringing forth peculiar phenomena, which we labeled as “complex experiences.”

Among these phenomena it would be possible to include the following complex experiences: complex thinking, flow experiences, the sublime, awe, Turning Points, body illusions, diversifying experiences, paradoxical experiences, expectation violations, transformation, and mental imagery.

Complex experiences can now be studied by means of sophisticated simulations able to act as a tradeoff between the uncontrollability of natural settings and the reductionism of the lab. It is the case of Virtual Reality—defined as 3D simulations—where it is also possible to interact with the objects present within the synthetic environment, thus leading to a sense of presence i.e., the illusion of being “there,” as if it was real life (Waterworth et al., 2015). However, there are also special conditions, such as light deprivation or blindness, which may be deemed as natural simulations of “as if” situations. Therefore, these complex phenomena range from an altered perception of one's own and others' bodies (Petkova and Ehrsson, 2008; Keizer et al., 2016) and powerful experiences based on the violation of laws of physics and logic (Ritter et al., 2012, 2014), to paradoxical situations almost impossible before the coming of new advanced technologies such as virtual reality, augmented reality, and mixed reality. Complex experiences are possible not only thanks to new technologies but especially through their intersection with artistic practices and storytelling. Indeed, the semiotic framework provided by art can be used to amplify the potential of these experiences. Within this arising framework, being “complex” has often been referred to as human experiences featured—at the emotional level—with several even conflicting nuances, often overlapping opposing components, - at the cognitive level—with a novel way of thinking based on complex systems' theory and schema violations, - at the perceptual level—with the ability to generate new states of consciousness or awareness regarding themselves, others, and the world, in individuals. Finally, at the overall level, all these components can be merged to generate an emerging phenomenon not easily separable in its subcomponents. This tentative definition can be considered as data driven, since, before collecting all the articles in this research topic, it would have been hard to accrue a sufficient number of recurrent features that could be associated with the same class of phenomena.

Indeed, the theoretical and methodological endeavor of researchers in both shaping new conceptual models, as well as in defining new methodological guidelines regarding these experiences, has been the main focus point of this research topic. With the hope to build a growing community of researchers around this editorial work, we systemized all the contributions by outlining where complexity dwells within all the works and phenomena investigated within this research topic. To this end, we discussed complexity associated with human experiences in terms of (i) emotional experiences including conflicting nuances and often overlapping opposing components, (ii) complex thinking and violations of basic worldviews, and (iii) overall experiences encompassing all the previous levels along with a perceptual level, which includes the ability to generate new states of consciousness or awareness regarding themselves, others, and the world, in individuals.

## Studying Emotional Experiences: a Story Still to be Written

Within this broad category, it would be possible to include, especially, all the studies focusing on complex emotional experiences (Grossmann et al., 2016; Berrios, 2019), such as awe and the sublime, as well as their impact on human behavior and cognition and those researches providing new methodological advancements for the study of emotions in general. The emotion of awe and the sublime are closely intertwined—if not overlapped—and their nature is far from being fully elucidated due to the complex interplay between negative and positive emotional subcomponents (Arcangeli et al.), along with the appraisal themes of vastness and the need for accommodation, which were suggested as underlying dimensions of both phenomena (Arcangeli et al.). Understanding of awe's complexity has been deepened by Chirico and Gaggioli, and it was featured as a long-lasting experience unfolding over time across different levels of complexity, from the electrical to the existential. Crucially, the key effects of awe have been indicated as potentially able to counteract some major symptoms of depression, such as hopelessness and rumination (Chirico and Gaggioli), given the transformative impact of this emotion, which emerged in the work of Sawada and Nomura. In this study, (positive) awe resulted as a driver of tolerance toward others' norm violations, thus hinting at a link with cognitive flexibility, another asset to modulate depressive symptoms (Soltani et al., 2013).

## Methodological Advancements in Studying Emotional Experiences

At the methodological level, Balsamo et al. proposed a new framework for validating emotional stimuli suitable for experimental testing, which takes into account the complex interplay between emotions and their evolutionary function (in terms of elicitors' relevance and salience), overcoming standard dimensional and discrete models. This bottom-up validation approach allowed the selection of a new set of emotional images as well as common stimuli metrics researchers can rely on, thus respecting the complex interplay between emotions and their implicit effects on cognition and behavior. Things get more complex when dealing with a special kind of emotional stimuli, that is, faces. This is the field of emotionally expressive face recognition, in which a recent study by Zsido et al. showed that both adults and children were able to detect happy faces significantly faster than angry or fearful faces. Crucially, children also took significantly less time to find angry faces compared to fearful ones. Moreover, while children were slower than adults in recognizing the emotional expressions of other children or adults (regardless of the emotion displayed), adults were more sensitive than children in detecting emotions on the faces of other peers (adults) vs. faces of children. These results also suggested the intervention of an implicit component, that is, meaning, in the perceiver of emotional facial expression, thus opening up further research questions concerning the developmental trajectory of this process.

## Developmental Trajectories in Worldviews and Complex Thinking

To approach a complex phenomenon—such as the typical co-existence of multiple perspectives, theories, and models occurring within the didactics of psychology—a new way of thinking relying on complexity theory can be applied (Harmat and Herbert). Complexity thinking adopts a wide range of methods to study complex phenomena (Davis and Sumara, 2014), and it has been recently proposed for mapping teaching practices concerning psychology within Swedish secondary schools (Harmat and Herbert). One of the major challenges emerging from this study concerned the transformation of theories into practice, which should be operated by the pupils according to their own experiences and always in relation to a meso level (i.e., teachers' practices and knowledge) until a macro societal level of analysis has been reached. This constant self-reflection should be at the base of self-knowledge as an emergent property of interactions between the micro-level (i.e., students' knowledge), meso level (i.e., teachers' knowledge), and the macro one (i.e., societal). Overall, this approach should help students to see beyond the fragmentation of theories and models within psychology and to capture the sight of the whole by integrating new knowledge into existing knowledge, and, if necessary, also to restructure their implicit assumptions of lay psychology, which should be replaced with new incoming information (Harmat and Herbert; Tulis, 2018).

Indeed, all individuals are characterized by their inner assumptions regarding themselves, other people, and the world. In this last case, these generalized beliefs about how the world works are called Primals or Primal world beliefs (Clifton et al., 2019). While past theories usually endorsed a retrospective account of primals as shaped by past experiences e.g., (Janoff-Bulman, 1989; Foa and Rothbaum, 2001), (Clifton), instead, supported an interpretative approach and reported preliminary evidence in favor of this latter theory, coming from his past works on Primals (Clifton et al., 2019). However, since the core question concerning the potential of specific experiences in shaping Primals or vice versa persisted, a novel model was developed—the Cube Framework—mapping human experiences on three continua: (i) chronic-acute, (ii) internal-external, and (iii) positive-negative. From the permutations of these three dimensions, the author derived eight types of experiences that are worth further investigation in relation to Primals: bad choices, good choices, bad habits, good habits, bad luck, good luck, bad times, and good times. The author concluded with some pessimism concerning the reliability of some natural life experiences in shaping Primals. In the following paragraph, we illustrated some studies investigating the transformative potential of some ecological experiences on specific cognitive and emotional processes, including some relatively stables ones (Chirico et al., submitted).

We concluded here, focusing on a specific population: the elderly. Special attention has been devoted to a pervasive experience—the use of internet—which resulted in participants being able to change their accustomed attitudes, perceptions, and beliefs toward their life, others, and the world. Individuals who spent more time on the Internet perceived more Online Social Support, but developed more negative evaluations regarding themselves (i.e., self-esteem) and regarding their own life (i.e., Satisfaction with life). Therefore, change in relatively stable attitudes, beliefs, and perceptions was seen to be possible across the entire life span. However, some core beliefs—which are usually more resistant—may need peculiar complex experiences to change.

## Complex Experiences in Naturalistic and Ecological Contexts

Awe and the sublime resulted as hardly definable emotions, and recently have been better described as experiences due to their inner complexity (Chirico and Gaggioli, 2018). This change was not required for another more frequent complex experience often labeled as “optimal,” which is flow. Flow can be defined as the optimal psychological state in which perceived challenges are perfectly in balance with perceived skills required to perform a task, with clear goals, a sense of altered time, loss of self-consciousness, and increased sense of control over the task, which provides unambiguous feedback and a sense of being fused with the task itself (Csíkszentmihályi, 1990; Csikszentmihalyi and Csikszentmihalyi, 1992). Flow can be improved and solicited by means of different practices—which usually require active involvement- but recently, recurrent Qigong meditative practices were shown to be able to increase flow intensity over time along with positive affect (Pölönen et al.).

There is another pervasive multifaceted experience deemed as complex, which concerns individual's ability to hear with their “mind's ear” while not physically present, that is, musical imagery. Cotter showed that by endorsing a complexity thinking approach—by integrating multiple theories, evidenced from different literatures on this topic—in order to capture the sight of the whole, was also possible to recognize the basic rules underlying this “system” as well as to capture all its nuances. Specifically, two dimensions were identified to explain mental control in music-imagery, namely, the extent to which this experience begun voluntarily, and the degree of control after the experience occurred.

On the other hand, some classes of complex experiences were shown to be rarer than flow, and usually occur in response to novel, unexpected events able to drag people far from their realm of normality. This is the case of Diversifying Experiences (DEs). A natural setting of occurrence of this class of experiences was provided by an artistic format called “Dialogue in the Dark” (DD), which was proposed in Milan to normal sighted people and concerns a path in the absence of light. The format seeks to answer the question of what it would be like to live as a blind person for some time. A recent study (Chirico et al.) aimed to test the transformative potential of this experience in terms of increase in creative thinking vs. an equivalent experience in a park. Although results did not show a significantly higher creative thinking ability in the group undergoing the DD (vs. control group in the park), this experience resulted as more impacting, and generated significantly more intense positive emotions than the control. A key variable missed in this study was the effect of time on creative performance.

In another recent study, the effect of time was, instead, taken into consideration in the domain of blindness (Rindermann et al.). Specifically, preliminary evidence showed that visually impaired children performed significantly better in working memory tasks compared to children without visual impairment, but worse in terms of verbal comprehension (Rindermann et al.). A condition of visual impairment, alike the deprivation of light, if protracted, may be considered as a specific natural complex experience whose transformative potential unveils in the cognitive domain of memory and language.

Finally, traumatic life events have been always considered as potentially transformative experiences able to re-shape individuals' mental schema and assumptions regarding the word, themselves, and the others. By means of Virtual Reality (VR) simulations, nowadays, it would be possible to help women who lost their child to manage their complex emotions in a safe and controlled environment. Specifically, Corno et al. proposed an intervention protocol for women who lost their child consisting of VR emotional environments featuring events with their various emotional state related to the loss of their child. The multifaceted emotional experience characterizing perinatal loss, thus, could be simulated, managed, and overcome within a safe, controlled setting, under the supervision of expert clinicians and professionals.

## Conclusions

These contributions enabled the outlining of an emerging perspective in which complexity can be seen as a general attitude to study long-standing constructs (e.g., emotions, musical imagery, and working memory) in a novel way, by looking at the whole picture instead of stopping at the fragmented existing literatures and methods. Then, complexity was included as an adjective qualifying some experiences, which were the main object of interest of this research topic. For instance, some emotions have already been deemed as complex by other scholars (Berrios, 2019), and some cognitive processes already relied on the complex system theory as a general framework (Harmat and Herbert). Other phenomena have been explicitly labeled as “complex” only within this RT for the first time, and the method adopted for their analysis drew from and advanced existing models and methodological approaches to create a new one (Balsamo et al.). Some boundaries to study complex experiences within social science have been drawn. However, more systematic works collecting all these types of experiences would still be required.

Specifically, we learnt that it is possible to “think” in a complex way—which does not mean think of something complicated, instead, it entails dealing with the multifaceted nature of a phenomenon avoiding excessive reductionism or determinism. Then, complexity, often, resulted as related to something relatively stable, such as our beliefs toward the world, which may be violated to generate a sort of “update” of the whole system. Sometimes, complexity unfolds from a mixed emotion to an optimal experience, and some other times, it requires something more disruptive or diversifying, stemming from a basic violation—such as leaving our routine in the absence of life—to something transformative, both in a positive and in a negative way, that is, a traumatic way. All these experiences can shape our view of the world across the lifespan, and also, elderly people are not exempt from these phenomena. Crucially, methods adopted to consider the complexity associated with a natural experience can reveal new ways to see long-lasting phenomena.

A natural question that could be asked is: what's next? We would say, a lot.

First, researchers should orient their attention toward a systematic analysis of the theoretical link between complexity and experience. If for the former scholars can rely on the works of the psychology of complexity, for the latter aspect, it would be reasonable to draw from philosophy, still, to nurture a more substantial dialogue with philosophers. Finally, the Cube framework looks like a promising lens to capture the inner transformative potential of some experiences.

Then, the concept of schema violation may need a more ecological paradigm to be investigated—such as some art installations reproducing paradoxical scenarios, like the Dialogue in the Dark or some conditions such as blindness—and more comprehensive measures to be analyzed, thus, primal world beliefs are already showing themselves to be a good candidate. Crucially, the design of complex experiences still occupied a small portion of this research topic. Nevertheless, this aspect is usually a driver for translating sophisticated models and theories into something accessible to all individuals. The applied side of complex experience is urgently needed.

Finally, if living deals with complexity, and if psychology—as a social and human science—deals with experiences, then it is urgent to reconcile complexity and experience even in science, since one cannot exist without the other.

## Author Contributions

AC wrote the first draft. AG supervised the entire process. AC and AG gave contributions on the future steps, rhetoric, and rational. Both authors have contributed to conceptualizing and writing this Editorial and approved this version of the manuscript.

## Funding

This work was partially supported by the Grant: Promoting Education of Scientific and Technological Societal Issues Through Sublime (PROMETHEUS) Cariplo: 2019–3536.

## Conflict of Interest

The authors declare that the research was conducted in the absence of any commercial or financial relationships that could be construed as a potential conflict of interest.

## Publisher's Note

All claims expressed in this article are solely those of the authors and do not necessarily represent those of their affiliated organizations, or those of the publisher, the editors and the reviewers. Any product that may be evaluated in this article, or claim that may be made by its manufacturer, is not guaranteed or endorsed by the publisher.

## References

[B1] Berrios R. (2019). What is complex/emotional about emotional complexity? Front. Psychol. 10:1606. 10.3389/fpsyg.2019.01606 31354593 PMC6639786

[B2] Chirico A. Gaggioli A. (2018). Awe: “More than a feeling”. Humanistic Psychol. 46, 274–280. 10.1037/hum0000098

[B3] Cipresso P. (2010). Modeling Emotions at the Edge of Chaos. From Psychophysiology to Networked Emotions. VDM Verlag Dr. Müller.

[B4] Clifton J. D. Baker J. D. Park C. L. Yaden D. B. Clifton A. B. Terni P.. (2019). Primal world beliefs. Psychol. Assess. 31:82. 10.1037/pas0000639 30299119

[B5] Csíkszentmihályi M. (1990). Flow: The Psychology of Optimal Experience. Harper Collins.

[B6] Csikszentmihalyi M. Csikszentmihalyi I. S. (1992). Optimal Experience: Psychological Studies of Flow in Consciousness. Cambridge University Press.

[B7] Davis B. Sumara D. (2014). Complexity and Education: Inquiries Into Learning, Teaching, and Research. Routledge.

[B8] Foa E. B. Rothbaum B. O. (2001). Treating the Trauma of Rape: Cognitive-Behavioral Therapy for PTSD. Guilford Press.

[B9] Grossmann I. Huynh A. C. Ellsworth P. C. (2016). Emotional complexity: clarifying definitions and cultural correlates. J. Pers. Soc. Psychol. 111:895. 10.1037/pspp0000084 26692354

[B10] Janoff-Bulman R. (1989). Assumptive worlds and the stress of traumatic events: applications of the schema construct. Soc. Cogn. 7, 113–136. 10.1521/soco.1989.7.2.113

[B11] Keizer A. van Elburg A. Helms R. Dijkerman H. C. (2016). A virtual reality full body illusion improves body image disturbance in anorexia nervosa. PLoS ONE 11:e0163921. 10.1371/journal.pone.0163921 27711234 PMC5053411

[B12] Petkova V. I. Ehrsson H. H. (2008). If I were you: perceptual illusion of body swapping. PLoS ONE 3:e3832. 10.1371/journal.pone.0003832 19050755 PMC2585011

[B13] Ritter S. M. Damian R. I. Simonton D. K. van Baaren R. B. Strick M. Derks J.. (2012). Diversifying experiences enhance cognitive flexibility. J. Exp. Soc. Psychol. 48, 961–964. 10.1016/j.jesp.2012.02.009

[B14] Ritter S. M. Kühn S. Müller B. C. Van Baaren R. B. Brass M. Dijksterhuis A. (2014). The creative brain: corepresenting schema violations enhances TPJ activity and boosts cognitive flexibility. Creat. Res. J. 26, 144–150. 10.1080/10400419.2014.901061

[B15] Soltani E. Shareh H. Bahrainian S. A. Farmani A. (2013). The mediating role of cognitive flexibility in correlation of coping styles and resilience with depression. Pajoohandeh J. 18, 88–96. Available online at: http://pajoohande.sbmu.ac.ir/article-1-1518-en.html

[B16] Tulis M. (2018). Da ist immer noch Luft drin! Zur Notwendigkeit einer didaktischen Konzeption kognitiver Umstrukturierungsprozesse im Psychologieunterricht, in Psychologiedidaktik und Evaluation XII, eds Krämer M. Preiser S. Brusdeylin K. (Aachen: Shaker Verlag).

[B17] Waterworth J. A. Waterworth E. L. Riva G. Mantovani F. (2015). Presence: form, content and consciousness, in Immersed in Media: Telepresence Theory, Measurement & Technology, eds Lombard B. F. Freeman M. IJsselsteijn J. Schaevitz W. (Berlin: Springer), 35–58. 10.1007/978-3-319-10190-3_3

